# Neuroinflammation induces synaptic scaling through IL-1β-mediated activation of the transcriptional repressor REST/NRSF

**DOI:** 10.1038/s41419-021-03465-6

**Published:** 2021-02-15

**Authors:** Federica Buffolo, Valentina Petrosino, Martina Albini, Matteo Moschetta, Federico Carlini, Thomas Floss, Nicole Kerlero de Rosbo, Fabrizia Cesca, Anna Rocchi, Antonio Uccelli, Fabio Benfenati

**Affiliations:** 1 Center for Synaptic Neuroscience and Technology, Istituto Italiano di Tecnologia, Largo Rosanna Benzi 10, 16132 Genova, Italy; 2 Department of Experimental Medicine, University of Genova, Viale Benedetto XV, 3, 16132 Genova, Italy; 3 Department of Neurosciences, Rehabilitation, Ophthalmology, Genetics, Maternal and Child Health, University of Genova, Largo P. Daneo, 3, 16132 Genova, Italy; 4 IRCCS, Ospedale Policlinico San Martino, Largo Rosanna Benzi 10, 16132 Genova, Italy; 5 Helmholtz Zentrum München, Deutsches Forschungszentrum für Gesundheit und Umwelt (GmbH), Ingolstädter Landstr. 1, 85764 Neuherberg, Germany; 6 Department of Life Sciences, University of Trieste, Trieste, 34127 Italy

**Keywords:** Cytokines, Cellular neuroscience

## Abstract

Neuroinflammation is associated with synapse dysfunction and cognitive decline in patients and animal models. One candidate for translating the inflammatory stress into structural and functional changes in neural networks is the transcriptional repressor RE1-silencing transcription factor (REST) that regulates the expression of a wide cluster of neuron-specific genes during neurogenesis and in mature neurons. To study the cellular and molecular pathways activated under inflammatory conditions mimicking the experimental autoimmune encephalomyelitis (EAE) environment, we analyzed REST activity in neuroblastoma cells and mouse cortical neurons treated with activated T cell or microglia supernatant and distinct pro-inflammatory cytokines. We found that REST is activated by a variety of neuroinflammatory stimuli in both neuroblastoma cells and primary neurons, indicating that a vast transcriptional change is triggered during neuroinflammation. While a dual activation of REST and its dominant-negative splicing isoform REST4 was observed in N2a neuroblastoma cells, primary neurons responded with a pure full-length REST upregulation in the absence of changes in REST4 expression. In both cases, REST upregulation was associated with activation of Wnt signaling and increased nuclear translocation of β-catenin, a well-known intracellular transduction pathway in neuroinflammation. Among single cytokines, IL-1β caused a potent and prompt increase in REST transcription and translation in neurons, which promoted a delayed and strong synaptic downscaling specific for excitatory synapses, with decreased frequency and amplitude of spontaneous synaptic currents, decreased density of excitatory synaptic connections, and decreased frequency of action potential-evoked Ca^2+^ transients. Most important, the IL-1β effects on excitatory transmission were strictly REST dependent, as conditional deletion of REST completely occluded the effects of IL-1β activation on synaptic transmission and network excitability. Our results demonstrate that REST upregulation represents a new pathogenic mechanism for the synaptic dysfunctions observed under neuroinflammatory conditions and identify the REST pathway as therapeutic target for EAE and, potentially, for multiple sclerosis.

## Introduction

RE1-silencing transcription factor (REST), also known as neuron-restrictive silencer factor (NRSF)^1^, is a transcriptional repressor that binds a specific 21 bp *consensus* sequence named repressor element 1 (RE-1)^2^. Through its N- and C-terminal domains, REST recruits a number of chromatin remodeling factors, ultimately mediating the transcriptional repression of target genes^3^. REST-binding sites have been identified in thousands of coding and non-coding sequences^4–8^. The repressive function of REST is relevant for central nervous system (CNS) physiology, as it modulates membrane excitability and synaptic transmission by controlling the expression of many presynaptic and postsynaptic proteins, such as ion channels^2,9,10^, neurotransmitter receptors^11–13^, and synaptic vesicle proteins^14,15^; see^16^ for review.

The human REST mRNA can be spliced into at least six alternative neuron- and disease-associated transcripts^17–19^. Among all these splice variants, the most studied is REST4, which lacks the critical domains required for transcriptional silencing. The biological function of REST4 is not completely understood. REST4 was initially shown to have repressor activity^17^; however, REST4 may also interact with REST preventing it from binding RE-1 sequences, thus causing de-repression^11–13,20^. REST4 levels increase in response to a variety of stimuli in neuronal cells, such as chronic stressful events^21^. REST4 expression is regulated by the neural-specific Ser/Arg repeat-related protein of 100 kDa (nSR100/SRRM4)^22,23^. The presence of distinct REST isoforms has been frequently overlooked in the literature; however, it has to be considered in order to avoid data misinterpretation^24^.

Thanks to its coordinated repressor activity on a large cluster of neural genes, REST is one of the main actors of homeostatic plasticity in neurons, fine-tuning intrinsic excitability and synaptic transmission in response to hyperexcitation through transient changes in its expression levels^25,26^. The expression and activity of REST are altered in a number of neurological diseases; depending on the tissue and the specific pathology, REST acts under some circumstances as a protective factor and under other conditions as a promoter of insult-induced neuronal death or dysfunction. For example, increased REST levels have been observed after epileptic or ischemic insults, but whether such increase is protective or deleterious is still not understood^27–31^. Recent evidence supports a protective role of REST in CNS diseases: REST is neuroprotective in aging and counteracts the development of Alzheimer’s disease by protecting neurons from oxidative stress and amyloid β-induced toxicity^32^. In addition, longevity in humans was recently found to be associated with REST upregulation and repression of excitation-related genes^33^.

Neuroinflammation is a common feature of several diseases. Indeed, neuroinflammation, mediated by peripheral cells infiltrating the CNS and resident immune cells such as microglia, is a key process in the complex biological response of the brain to insults and influences the outcome and the severity of the CNS pathology. The overall effect of neuroinflammation is the result of the action of a wide array of factors including reactive oxygen species, cytokines, chemokines, and growth factors, all of which may exert either neuroprotective or neurotoxic effects. In multiple sclerosis (MS), an autoimmune disease of the CNS, neuroinflammation is triggered by the infiltration of immune cells, in particular autoreactive T and B cells and macrophages, followed by a broader neurodegenerative process^34,35^. In the animal model for MS, experimental autoimmune encephalomyelitis (EAE), induced by the immunization of susceptible animals with CNS antigens^36,37^, infiltrating immune cells interacting with activated CNS-resident microglia and astrocytes promote demyelination, resulting in neuro-axonal injury. These events occur through both direct cell–cell contacts and the release of soluble inflammatory and neurotoxic mediators^38^. Activated microglia cells can release different kinds of mediators, including both neurotoxic and neurotrophic molecules, pro- and anti-inflammatory cytokines^39^, in particular interleukin (IL)-1β, tumor necrosis factor (TNF)-α, and chemokines^40^. Infiltrating T cells are also responsible for the direct release of pro-inflammatory cytokines such as granulocyte macrophage colony-stimulating factor^41^, interferon (IFN)-γ^42^, IL-17^43^, and IL-22^44^, which are crucial to disease worsening. In addition to the in vivo animal models, in vitro neuronal cultures are also used to analyze axonal damage and neurodegeneration induced upon inflammatory insults. In this context, neuronal cultures can be used to study the effect of cytokines that are present in the neuroinflammatory milieu of CNS diseases^45–48^.

Despite the extensive knowledge about the involvement of REST in several CNS diseases, whether changes in its expression/activity are specifically associated with neuroinflammation is still poorly understood. In this work, we used two in vitro models i.e., murine neuroblastoma cells (N2a) and mouse primary neurons, which were challenged with either the supernatant of inflammatory activated T cells^49–51^ and microglia or with specific pro-inflammatory cytokines, to investigate the changes in expression and activity of REST and its splice isoform REST4 under inflammatory conditions and the cellular mechanisms leading to such changes. We found that REST and REST4 are differentially regulated in different cell models, through a mechanism involving the activation of the Wnt/β-catenin pathway. Moreover, in primary neurons, inflammatory stimuli induce synaptic scaling specifically through IL-1β-mediated activation of REST. Altogether, our data support the idea that REST is an important player in the inflammatory process, possibly representing a valid target to treat neurological illnesses characterized by inflammation.

## Materials and methods

### Preparation and treatment of N2a cultures

N2a cells were cultured in Dulbecco’s Modified Eagle Medium (DMEM) supplemented with 10% fetal bovine serum (FBS), glutamine (2 mM), and antibiotics (penicillin and streptomycin, 10,000 U/ml), in a humidified 5% CO_2_ atmosphere at 37 °C. All reagents for in vitro experiments were purchased from Gibco (Thermo-Fisher Scientific, Milano, Italy). N2a cells were seeded in 24-well plates (3 × 10^5^) or 6-well plates (1 × 10^6^) in 1 or 2 ml of DMEM. N2a cells were stimulated with supernatant from activated splenocytes (see below) at a volume ratio of 1:3 for different times. For differentiation, N2a cells were seeded at 60% confluence, and DMEM was replaced after 24 h with differentiating medium composed of DMEM containing 1% FBS and 20 μM retinoic acid. Differentiating medium was replaced daily for 3 days, after which the cells were used for the experiments.

### Preparation of activated T cell supernatant

Splenocyte suspensions were prepared by mechanically teasing the spleen of naive mice with the blunt end of a 10-ml plastic syringe plunger in a Petri dish, then passing suspensions through a BD Falcon 70 μm cell strainer (Corning, Glendale, AZ, US) to remove debris, and finally suspending the cells in RPMI 1640 (Thermo-Fisher Scientific) supplemented with 10% FBS, glutamine (2 mM), antibiotics (penicillin and streptomycin, 10,000 U/ml), and 50 μM β-mercaptoethanol. Splenocytes were cultured (1 × 10^6^ cells/well) in 24-well plates for 48 h in the presence or absence of anti-CD3/CD28 antibodies (clones 17A2 and 37.51, respectively, Biolegend, San Diego, CA, US) at a concentration of 1 µg/ml for each antibody, as indicated by the manufacturer. Supernatants were collected and stored frozen at −20 °C until use.

### Preparation and treatments of primary neurons

All experiments were carried out in accordance with the guidelines established by the European Community Council (Directive 2010/63/EU of 22 September 2010) and were approved by the local Ethical Committee and the Italian Ministry of Health. Efforts were made to minimize suffering and reduce the number of animals used. Primary cortical cultures were prepared from wild-type C57BL/6J mice (Charles River Laboratories, Calco, Italy) at embryonic day 17 as described^31^. Postnatal cortical neurons were prepared from P0 to P1 pups from the previously described REST^GTi^ mice^52^. Pups were decapitated, cortices were removed, and enzymatically dissociated. Cortical neurons were plated on poly-L-lysine (PLL)-coated (0.1 mg/ml) glass coverslips (Thermo-Fisher Scientific) at a density of 40,000 cells/ml for immunocytochemical experiments or 250,000 cells/ml for western blot experiments and maintained up to 14 days in vitro (DIV) at 37 °C, 5% CO_2_, 95% humidity in a culture medium consisting of Neurobasal Medium for embryonic neurons or Neurobasal A Medium for postnatal neurons, B-27 (2%), GlutaMAX (1%), and penicillin/streptomycin (1%; Thermo-Fisher Scientific). In the experiments involving exposure of neurons to supernatant derived from activated T cells, cultures were incubated with supernatant derived from activated T cells. Controls were subjected to the same medium change with the addition of equivalent volume of supernatant derived from inactivated T cells. Supernatants were added at 7 DIV and cells were collected at 8 DIV. In the experiments involving pharmacological treatments, cultures were incubated with a medium containing pro-inflammatory cytokines, alone or in combination: IL-6 (20 ng/ml), TNF-α (20 ng/ml), IL-1β, (20 ng/ml), IFN-γ (20 ng/ml) (PeproTech Inc., Rocky Hill, NJ, USA). Controls were subjected to the same medium change with the addition of equivalent volumes of 0.1% bovine serum albumin (BSA; Sigma) in H_2_O. Drugs were added at different time points and cells were collected at 7 or 14 DIV. The production of VSV-pseudotyped third-generation lentiviral particles was performed as described^53^. pLenti-PGK-Cre-EGFP or pLenti-PGK-ΔCre-EGFP plasmids were obtained as previously described^54^. Primary neurons were infected at 1 DIV at a multiplicity of infection of 10. After 24 h, half of the medium was replaced with fresh medium.

### Microglia–neuron co-cultures

Microglial cultures were prepared from 18-day mixed primary glial cultures obtained from the cerebral cortex of C57BL6/J pups at postnatal day 1, as previously described^55^ with minor modifications. Briefly, cortices were collected, washed, and minced in KRB buffer containing 70.7 g/l NaCl, 3.6 g/l KCl, 1.66 g/l KH_2_PO_4_, 21.4 g/l NaHCO_3_, 25.7 g/l glucose, phenol red supplemented with 6 g/l BSA, and 0.06% MgSO_4_ in endotoxin-free water. Cortices were digested in 25 mg/ml trypsin (Sigma) for 15 min at 37 °C with gentle shaking. The digestion was stopped by adding 0.26 mg/ml trypsin inhibitor and 0.12 mg/ml DNase. Tissue was mechanically dissociated and the cell suspension was washed once in KRB buffer supplemented with 0.09% MgSO_4_ and 0.00144% CaCl_2_. Glia cells were cultured in PLL-coated flasks in DMEM supplemented with 10% FBS, 1 mM glutamine, penicillin, and streptomycin (Gibco). After 18 DIV, microglial cells were harvested from the mixed primary glial cultures by mild shaking, resuspended in serum-free DMEM, and plated on plastic wells or coverslips coated with growth factor-reduced matrigel (BD) at a density of 50,000 cells/cm^2^. Microglial cells were seeded on matrigel-coated polyethylene tetraphthalate membranes etched with 3 μm pores (Corning)^53^. On day 1, cells were incubated for 24 h with lipopolysaccharide (LPS, 0.1 µg/ml; L2654, Sigma-Aldrich) or vehicle. After incubation, microglia-coated transwells were then placed into plates with 14 DIV cultured neurons. All experiments occurred immediately following 24 h in co-culture.

### RNA isolation and real-time PCR analysis

Total cellular RNA was extracted using Trizol (Qiagen, Hilden, Germany) and RNeasy MinElute Cleanup Kit (Qiagen), and cDNA was synthesized starting from 0.5 µg of RNA, using the SuperScript IV Reverse Transcriptase Kit (Thermo-Fisher) and following the manufacturer’s instructions. The cDNA was amplified and quantified by quantitative real-time PCR with the SYBR Green Master Mix (Qiagen) and Bio-Rad (Hercules, CA, US) CFX96 Real-Time PCR Detection System with:

REST primers: [5’-ACCACTGGAGGAAACACCTG-3’ (sense) and 5’-ATGGCTTCTCACCTGAATGAGTC-3’ (antisense)];

REST4 primers: [5’-ACCACTGGAGGAAACACCTG-3’ (sense) and 5’-CTCACCCAGCTAGATCACACTC-3’ (antisense)];

Scn2a primers [5’-GGCTCTGCTGTCATTGTTGGTA-3’ (sense) and 5’-GAAGGCTAGGTGAGTACATCCC-3’ (antisense)];

Syn1 primers: [5’-ATCTTCCTCCAACCTCCA-3’ (sense) and 5’-TTTGCTTCCCGACTCTTC-3’ (antisense)].

Transcript levels from each sample were normalized to Actin primers [5’-AAGTGGTTACAGGAAGTCC-3’ (sense) and 5’-ATAATTTACACAGAAGCAATGC-3’ (antisense)], Gapdh primers [5’-GAACATCATCCCTGCATCCA-3’ (sense) and 5’-CCAGTGAGCTTCCCGTTCA-3’ (antisense)], and Hprt1 primers [5’-AAGCTTGCTGGTGAAAAGGA-3’ (sense) and 5’-TTGCGCTCATCTTAGGCTTT -3’ (antisense)].

### Western blotting analysis

Cells were lysed in RIPA buffer (10 mM Tris-HCl pH 7.4, 140 mM NaCl, 1 mM EDTA, 0.5 mM EGTA, 1% Triton X-100, 0.1% sodium dodecyl sulfate (SDS), 0.1% sodium deoxycholate) supplemented with proteases and phosphatases inhibitors (complete EDTA-free protease inhibitors, Roche Diagnostic (Risch-Rotkreuz, Switzerland), serine/threonine phosphatase inhibitor, and tyrosine phosphatase inhibitor (Sigma Aldrich, Milano, Italy) and equal amounts of proteins were loaded, as determined by BCA assay (Thermo-Fisher Scientific). Samples were separated on 6–10% SDS polyacrylamide gels and proteins transferred to a nitrocellulose membrane with 0.2-μm pore size (GE Healthcare, Chicago, IL, US). Membranes were washed in TBS containing 0.1% Tween (TBST) and blocked with 5% BSA in TBST buffer for 1 h at room temperature (RT). Primary antibodies were diluted in blocking solution and incubated overnight at 4 °C in a humidified chamber. Primary antibodies used were: anti-REST 1:1000 (#07–579, Merck-Millipore, Milano, Italy), anti-Calnexin 1:70,000 (#ADI-SPA-860, Enzo Life Sciences, Farmingdale, NY, US), anti-REST4 1:1000 (homemade, kindly gifted by Dr. Uchida, Yamaguchi University Graduate School of Medicine), anti-phospho Ser133 CREB 1:500 (#87G3, Cell Signaling, Danvers, MA, US), and nSR100 1:1000 (#PA5–45083, Thermo-Fisher Scientific). Membranes were washed 3 times in TBST to eliminate primary antibody in excess. Appropriate secondary horseradish peroxidase-conjugated antibodies were diluted in blocking solution and incubated for 1 h at RT. Membranes were washed three times in TBST to remove secondary antibodies in excess and detected using the ECL™ Western Blotting Detection Reagents (GE Healthcare BioSciences, Buckinghamshire, UK). Images were acquired via the ChemiDoc MP System (Bio-Rad).

### Subcellular fractionation

Nuclear and cytosolic fractions were separated as previously described^56^. Briefly, cells were washed with phosphate-buffered saline and harvested with buffer A (10 mM HEPES pH 7.9, 50 mM NaCl, 0.5 M Sucrose, 0.1 mM EDTA, 0.5% Triton X-100, 1 mM dithiothreitol (DTT) with fresh protease/phosphatase inhibitors). Cells were filtered with a syringe and centrifuged at 3000 × *g* for 2 min. The sedimented crude nuclei were washed twice with buffer A (10 mM HEPES pH 7.9, 10 mM KCL, 0.1 mM EDTA, 0.1 mM EGTA, 1 mM DTT with protease/phosphatase inhibitors) and resuspended in buffer C (10 mM HEPES pH 7.9, 500 mM NaCl, 0.1 mM EDTA, 0.1 mM EGTA, 0.1% NP-40 1 mM DTT with protease/phosphatase inhibitors). The suspension was rotated for 15 min at 4 °C, sonicated, and clarified by centrifugation at 18,000 × *g* for 15 min. The purity of the fractions was checked by using antibodies to the cytosolic marker GAPDH (14C10, #2118, Cell signaling) and the nuclear marker Lamin B1 (NBP1–19804, Novus Biologicals).

### Patch-clamp electrophysiology

Primary mouse cortical neurons incubated under the various conditions were used for patch-clamp recordings at 14 DIV. All experiments were performed using an EPC-10 amplifier controlled by the PatchMaster software (HEKA Elektronik, Lambrecht/Pfalz, Germany) and an inverted DMI6000 microscope (Leica Microsystems GmbH). Patch electrodes fabricated from thick borosilicate glasses were pulled to a final resistance of 4–5 MΩ. Recordings with leak current >100 pA were discarded. The standard Tyrode’s extracellular solution contained (in mM): 140 NaCl, 4 KCl, 2 MgCl_2_, 2 CaCl_2_, 10 HEPES, 5 glucose, pH 7.4, with NaOH and osmolarity adjusted to ~315 mOsm/l with mannitol. The intracellular (pipette) solution was composed of (in mM): 126 K gluconate, 4 NaCl, 1 MgSO_4_, 0.02 CaCl_2_, 0.1 BAPTA, 15 glucose, 5 Hepes, 3 ATP, and 0.1 GTP, pH 7.3. Experiments were carried out at RT (20–24 °C). All parameters were analyzed using the Minianalysis program (Synaptosoft, Leonia, NJ, USA) and Prism7 (GraphPad Software, Inc.) software. Miniature postsynaptic currents (mPSCs) were recorded in voltage-clamp configuration at −70 mV of membrane potential in the presence of tetrodotoxin (TTX, 300 nM) in the extracellular solution to block the generation and propagation of spontaneous action potentials. To isolate excitatory mPSCs, D-(−)-2-amino-5-phosphonopentanoic acid (D-AP5; 50 μM), bicuculline methiodide (30 μM), and (2 S)-3-[[(1S)-1-(3,4-dichlorophenyl)ethyl]amino-2-hydroxypropyl] (phenylmethyl)phosphinic acid hydrochloride (CGP58845; 5 μM) were added to block *N*-methyl-d-aspartate (NMDA), GABA_A_, and GABA_B_ receptors, respectively. To isolate inhibitory mPSCs, D-AP5 (50 mM) and 6-cyano-7 nitroquinoxaline-2,3-dione (10 μM) were added to block NMDA and non-NMDA, respectively. All reagents were purchased from Sigma Aldrich or Tocris (Tocris, Avonmouth, Bristol, UK).

### Calcium imaging

Cells were loaded with 1 μg/ml cell-permeable Fura-2 AM (#F1221, ThermoFisher) in the culture medium and maintained for 30 min in the incubator. Cells were then washed with culture medium and incubated for 30 min to allow hydrolysis of the esterified groups. Coverslips with cells were mounted on the imaging chamber and loaded with 0.5 ml of culture medium. Fura-2-loaded cultures were observed with an IX-81 motorized inverted epifluorescence microscope (Olympus, Tokyo, Japan) using a UplanSAPO ×63 1.35 NA oil-immersion objective (Olympus), and recordings were performed from visual fields containing 8 ± 3 infected neurons on average. Samples were excited at 340 and 380 nm by an MT20 Hg–Xe lamp (Olympus). The exciting light was separated from the emitted light using a 395-nm dichroic mirror. Images of fluorescence emission >510 nm were acquired continuously for a maximum of 20 min (200 ms single exposure time) by using a Hamamatsu Orca-ER IEEE1394 CCD camera (Hamamatsu Photonics, Hamamatsu City, Japan). The camera operated on 2 × 2 pixel-binning mode, and the imaging system was controlled by an integrating imaging software package (Cell^∧^R; Olympus). During the analysis, cells were selected by drawing regions of interest around their bodies to reduce the background. Traces were obtained from 340/380 ratio. Peaks with at least 2% of difference with respect to the baseline were considered and their frequency was calculated as the total number of peaks over the recording time.

### Cell viability

Mouse cortical neurons were seeded on glass coverslips for 14 days. Samples were treated with either vehicle (0.1% BSA) or IL-1β (20 ng/ml) for 20 min at 7 DIV and analyzed at 14 DIV. Cells were live stained for 3 min at RT with propidium iodide (PI; 1 μM) for cell death quantification, fluorescein diacetate (2 μM) for cell viability, and Hoechst 33342 (1 μM) for nuclear counting. Cell viability was quantified at ×10 magnification using a Nikon Eclipse-80i upright epifluorescence microscope (Nikon, Tokyo, Japan) with random sampling of at least 5 fields per sample (*n* = 8 samples, from 3 independent cell preparations). Cell death values were obtained from the ratio of PI/Hoechst 33342-positive cells and were normalized to the values of vehicle-treated samples. Image analysis was performed using the ImageJ software and the Cell Counter plugin.

### Immunofluorescence

Cells were stained as described^53^. The following primary antibodies were used: anti-vesicular glutamate transporter-1 (VGLUT1, #135 304; Synaptic Systems, Gottingen, Germany), anti-Homer (#160011; Synaptic Systems), anti-vesicular GABA transporter (VGAT; #131003, Synaptic System), anti-Gephyrin (#147011; Synaptic System), anti-β-catenin (#PA5–19469; Thermo-Fisher Scientific), and guinea pig anti-Iba1 (#234004; Synaptic Systems). Fluorescently conjugated secondary antibodies were from Molecular Probes (Thermo-Fischer Scientific). Image acquisitions were performed using a confocal microscope (SP8, Leica Microsystems, Wetzlar, Germany) at ×63 (1.4 NA) magnification. Z-stacks were acquired every 300 nm, 10 fields/sample. 4,6-Diamidino-2-phenylindole staining was used to stain nuclei. The colocalization analysis was performed by evaluating the labeling of the VGLUT1/Homer1 or VGAT/Gephyrin synaptic protein couples. Co-localization puncta with areas of 0.1–2 mm^2^ were considered bona fide synaptic boutons. Synaptic boutons along neurites were manually counted on 30 μm stretches starting from the cell body.

### Statistical analysis

Results are shown as mean ± sem. Normal distribution of data was assessed using Kolmogorov–Smirnov test. The two-tailed unpaired Student’s *t* test was used to compare two normally distributed sample groups, while either one- or two-way analysis of variance followed by Bonferroni’s multiple comparison test was used to compare more than two normally distributed sample groups. For datasets of non-normal distribution, Mann–Whitney *U* test or Kruskal–Wallis/Dunn’s tests were used. A *p* value <0.05 was considered significant. Statistical analysis was carried out using SigmaStat 13 (Systat Software).

## Results

### Exposure to supernatant from activated T cells differentially regulates REST isoform expression in N2a cells and primary cortical neurons

**Fig. 1:**
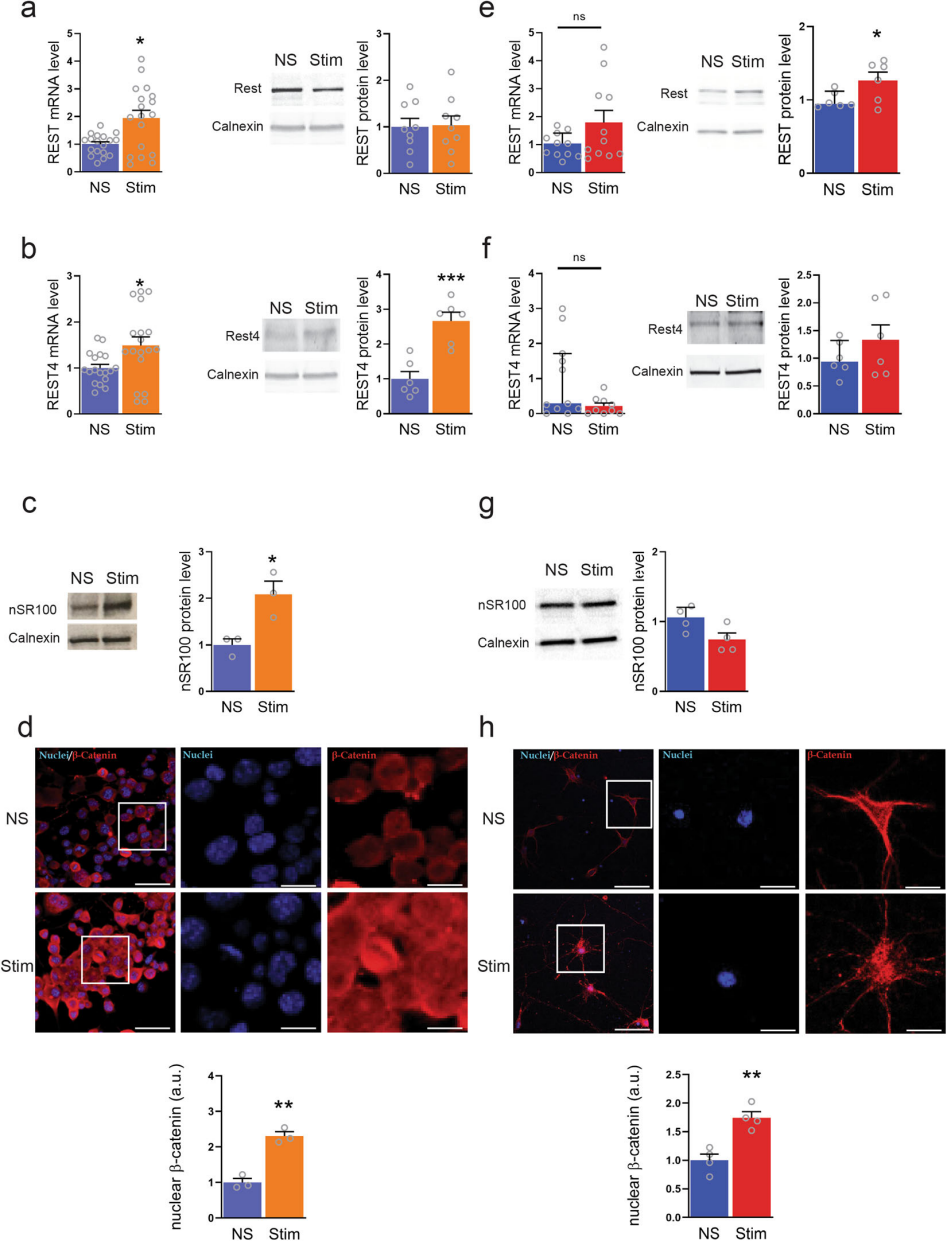
Exposure to activated T cell supernatant differentially regulates alternative splicing of REST in N2a cells and primary cortical neurons. **a**–**d** Differentiated N2a cells were exposed to either activated (Stim) or non-activated (NS) T cell supernatant for 24 h. **a**, **b** qRT-PCR (left) and western blotting analysis (right) were used to assess full-length REST (**a**) and REST4 (**b**) expression. **c**, **d** Analysis of nSR100 (**c**) and nuclear β-catenin (**d**) signals in Stim N2a cells compared to the control condition. **e**–**h** The same experimental procedures were carried out for primary cortical neurons. **e**, **f** qRT-PCR (left panels) and western blotting analysis (right panels) were used to assess REST (**e**) and REST4 (**f**) expression. **g**, **h** nSR100 (**g**) and nuclear β-catenin (**h**) signals in Stim neurons as compared to the control condition. Gapdh, Actin, and Hprt1 were used as housekeeping genes in qRT-PCR analyses. Calnexin was used as loading control for western blotting analyses. **d**, **h** Analysis of nuclear β-catenin signal. Representative images (top) and quantification (bottom) of nuclear β-catenin immunoreactivity (red) in N2a cells (**d**) and primary cortical neurons (**h**) treated with either control supernatant (NS) or activated T cell supernatant (Stim) for 24 h. DAPI-stained nuclei are shown in blue. Separate channels are shown for the high-magnification images of the boxed regions. Scale bars, 50 and 10 μm for low and high magnification, respectively. Bars show mean ± sem of at least *n* = 3 independent experiments with individual data points. *p < 0.05, **p < 0.01, ***p < 0.001, ns: *p* > 0.05; Mann–Whitney *U* test/unpaired two-tailed Student’s *t* test.

To address the changes in REST expression levels induced by a pro-inflammatory environment, we exposed differentiated N2a cells and primary neurons to supernatant derived from activated T cells (see “Methods” for details) for 24 h. As a parallel control, cells and neurons were exposed to supernatant from non-activated T cells (Fig. 1). In N2a cells, the treatment induced an increase in REST and REST4 mRNA levels, with a concomitant increase of REST4 protein expression (Fig. 1a, b). Consistent with the transcriptional activation of REST4, a significantly increased expression of nSR100, one of the factors involved in regulating REST/REST4 splicing^23^, was observed (Fig. 1c). Interestingly, primary neurons showed a distinct transcriptional response: exposure to supernatant from activated T cells induced a trend for an increase in full-length REST mRNA that was associated with a significant increase in REST protein expression (Fig. 1e), in the absence of changes in the expression of REST4 at both mRNA and protein levels (Fig. 1f) and with unaltered nRS100 protein levels (Fig. 1g).

Wnt/β-catenin signaling, which is activated during neuroinflammatory processes^57^, is known to promote REST gene transcription^58^. Accordingly, we assessed the extent of β-catenin activation by monitoring β-catenin translocation into the nucleus under the two experimental conditions. Upon treatment with supernatant from activated T cells, nuclear β-catenin levels increased in both N2a cells and primary neurons, as compared to control conditions (Fig. 1d, h).

### Exposure to stimulated microglia-conditioned medium increases REST expression in primary cortical neurons

**Fig. 2:**
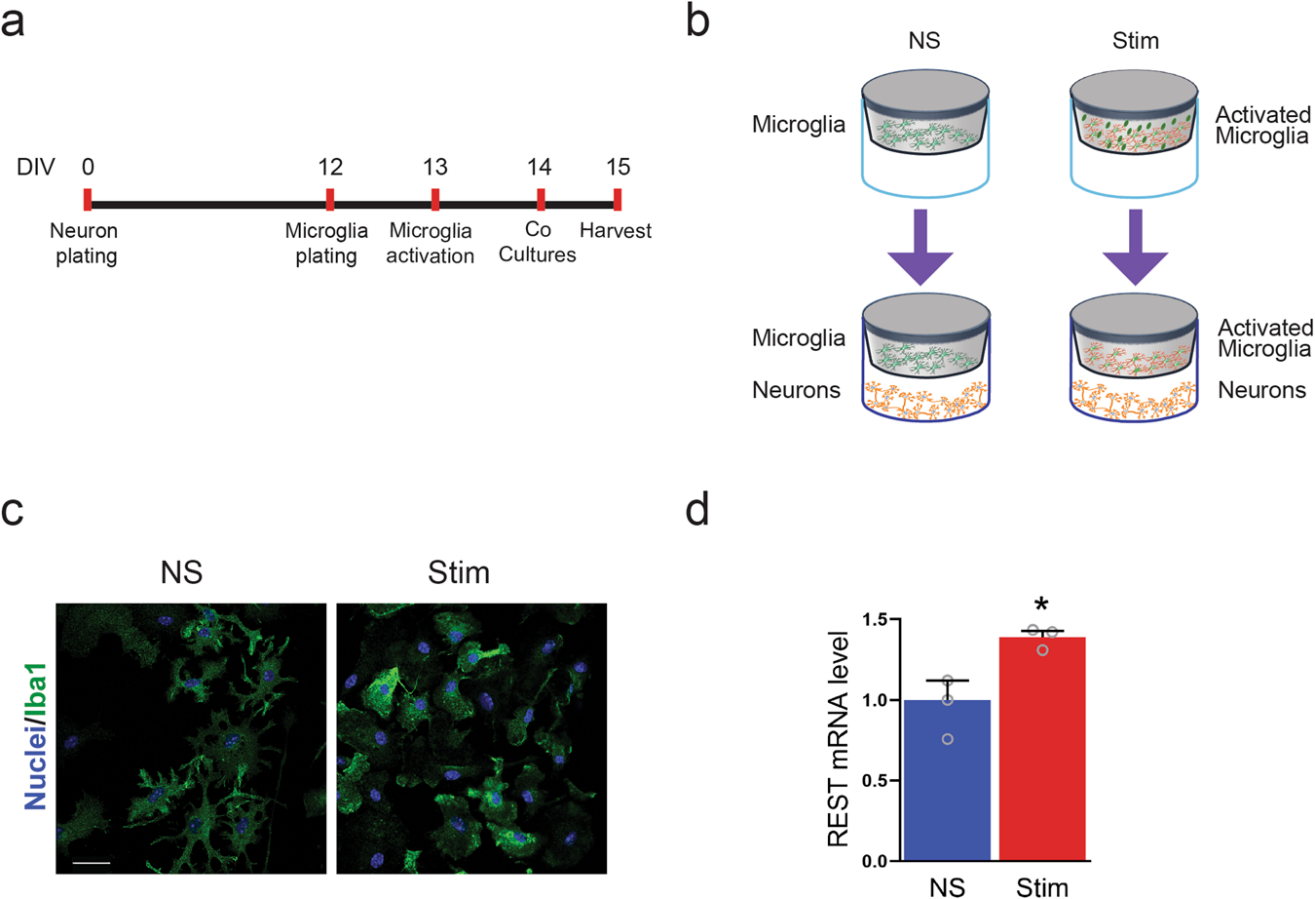
Contactless co-cultures of neurons and stimulated microglia increases the neuronal expression of REST. **a**, **b** Timeline (**a**) and schematics (**b**) of the experimental procedures for the contactless microglial/neuronal co-cultures. Primary microglial cells were harvested from the mixed primary glial cultures at 18 DIV and seeded on matrigel-coated Transwells^TM^ for 1 day before being treated for 24 h with either LPS (Stim) or vehicle (NS). After treatment, microglia-coated transwells were added to 14 DIV primary cortical neurons that were harvested 24 h later. **c** Microglial activation was verified by staining with the microglial marker Iba1. DAPI-stained nuclei are shown in blue. Scale bar, 25 µm. **d** After 24 h of contactless co-culture with either control or activated microglia, Transwells^TM^ were removed and neurons were harvested and subjected to qRT-PCR to assess the full-length REST expression. Gapdh, Actin, and Hprt1 were used as housekeeping genes. Bars show mean ± sem with superimposed individual points obtained from three independent preparations. Similar to activated T cell supernatant, the secretory activity of activated microglia increases REST expression in the co-cultured neurons. *p < 0.05; Mann–Whitney *U* test.

A prominent role for the release of pro-inflammatory cytokines in the brain is played by activated microglia. To ascertain that also microglial cytokines induce an activation of REST, as shown by treatment with activated T cell supernatant, we made contactless microglia–neuron cocultures in Transwells^TM^. Using this procedure, the neuronal medium can be conditioned by factors secreted by activated microglia in the absence of direct cell–cell contacts between microglia and neurons. Primary microglial cells on transwells were activated by exposure to LPS for 24 h, as shown by increased Iba1 immunoreactivity, and positioned on top of a 14 DIV primary neuronal culture for additional 24 h before harvesting neurons for the determination of REST mRNA levels (Fig. 2a–c). The data show that indeed the secretory activity of activated microglia brought about an increase in REST expression in the underlying neurons (Fig. 2d), suggesting that REST is also a target of the pro-inflammatory microglia secretome, of which IL-1 β and TNF-α are two of the main components.

### Expression of REST is selectively regulated by IL-1β in primary cortical neurons

**Fig. 3:**
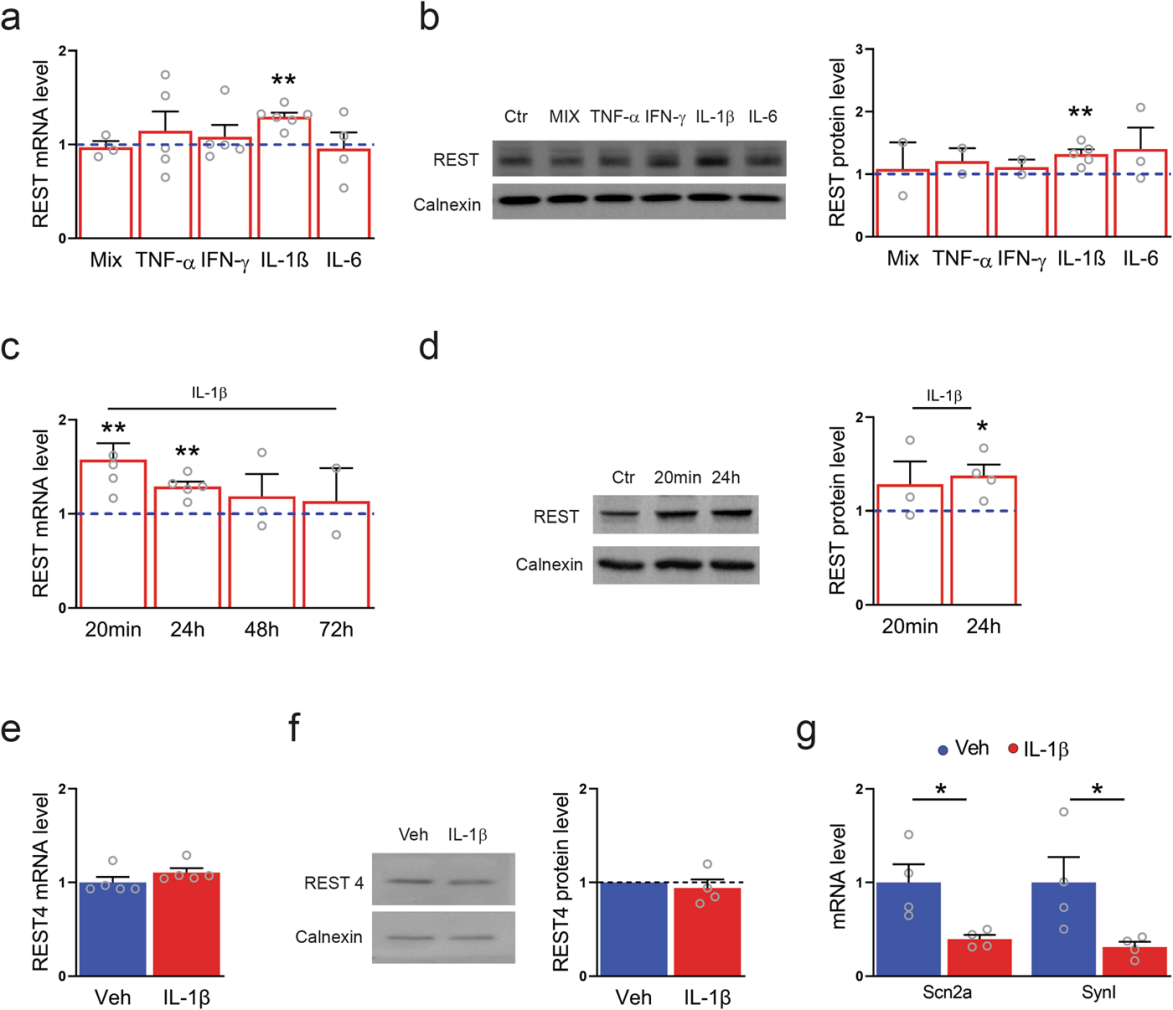
Expression of REST is selectively regulated by IL-1β in primary cortical neurons. **a** qRT-PCR analysis of REST mRNA levels upon treatment with the indicated pro-inflammatory cytokines for 24 h. **b** Representative immunoblot (left) and corresponding quantification (right) of REST protein levels under the same experimental conditions. **c** qRT-PCR analysis of REST mRNA levels upon IL-1β treatment for various times, as indicated. **d** Representative immunoblot (left) and corresponding quantification (right) of REST protein levels upon treatment with IL-1β for 20 min and 24 h, as compared to control condition. **e**, **f** qRT-PCR analysis (**e**) and immunoblotting (**f**) of REST4 upon exposure to either IL-1β or vehicle (Veh) for 24 h. **g** The mRNA levels of the Na^+^ channel Na_V_1.2 (Scn2a) and synapsin I (SynI) were quantified by qRT-PCR in IL-1β-treated neurons and compared to control. Gapdh, Actin, and Hprt1 were used as housekeeping genes in qRT-PCR analyses. Calnexin was used as loading control for western blotting analyses. Bar graphs show mean ± sem of at least *n* = 2 independent experiments with superimposed individual points. *p < 0.05, **p < 0.01; one-way ANOVA/Bonferroni’s tests (**a**–**d**); unpaired two-tailed Student’s *t* test (**e**–**g**).

To identify the pro-inflammatory molecule(s) responsible for the observed changes in REST expression in neurons, 7 DIV primary mouse cortical neurons were exposed for 24 h to specific pro-inflammatory cytokines (TNF-α, IL-1β, IFN-γ, IL-6, and a mix of all of them, 20 ng/ml) and REST expression was assessed at the mRNA and protein levels. Among the various treatments, REST mRNA and REST protein were significantly upregulated selectively upon IL-1β treatment in 7 DIV neurons, mimicking the effect of treatment with medium derived from activated T cells or activated microglia (Fig. 3a, b). IL-1β, expressed mostly by dendritic cells, macrophages, and activated microglia, all involved in the inflammatory response in EAE, is one of the most important mediators of the inflammatory response and modulates some of the inflammation-induced alterations of synaptic plasticity and structure^59^. In consideration of the fast kinetics through which IL-1β exerts its neuroprotective and neurotoxic actions^60^, a time response curve of REST mRNA and protein was performed by treating 7 DIV neurons with IL-1β (20 ng/ml) for various times ranging from 20 min to 72 h. REST transcription markedly increased at shorter times (20 min, Fig. 3c), while 24 h were needed to detect an effect at the translational level (Fig. 3d). On the contrary, exposure to IL-1β did not alter either REST4 mRNA or REST4 protein levels (Fig. 3e, f). In line with the selective upregulation of full-length REST, the mRNA levels of two well-known REST target genes, namely, the sodium channel Na_V_1.2 (Scn2a) and synapsin I (SynI)^15^, were decreased (Fig. 3g). Similar to what we observed with supernatant derived from activated T cells, treatment with IL-1β induced an increase in both REST and REST4 mRNAs in N2a cells, suggesting that in less differentiated neuronal-like cell lines both REST and REST4 are downstream players of IL-1β signaling (Supplementary Fig. 1), while the activation of REST/REST4 splicing and the expression of REST4 is lost in primary neurons.

### IL-1β increases β-catenin nuclear translocation and CREB activation and induces an impairment of excitatory synaptic strength in cortical neurons

**Fig. 4:**
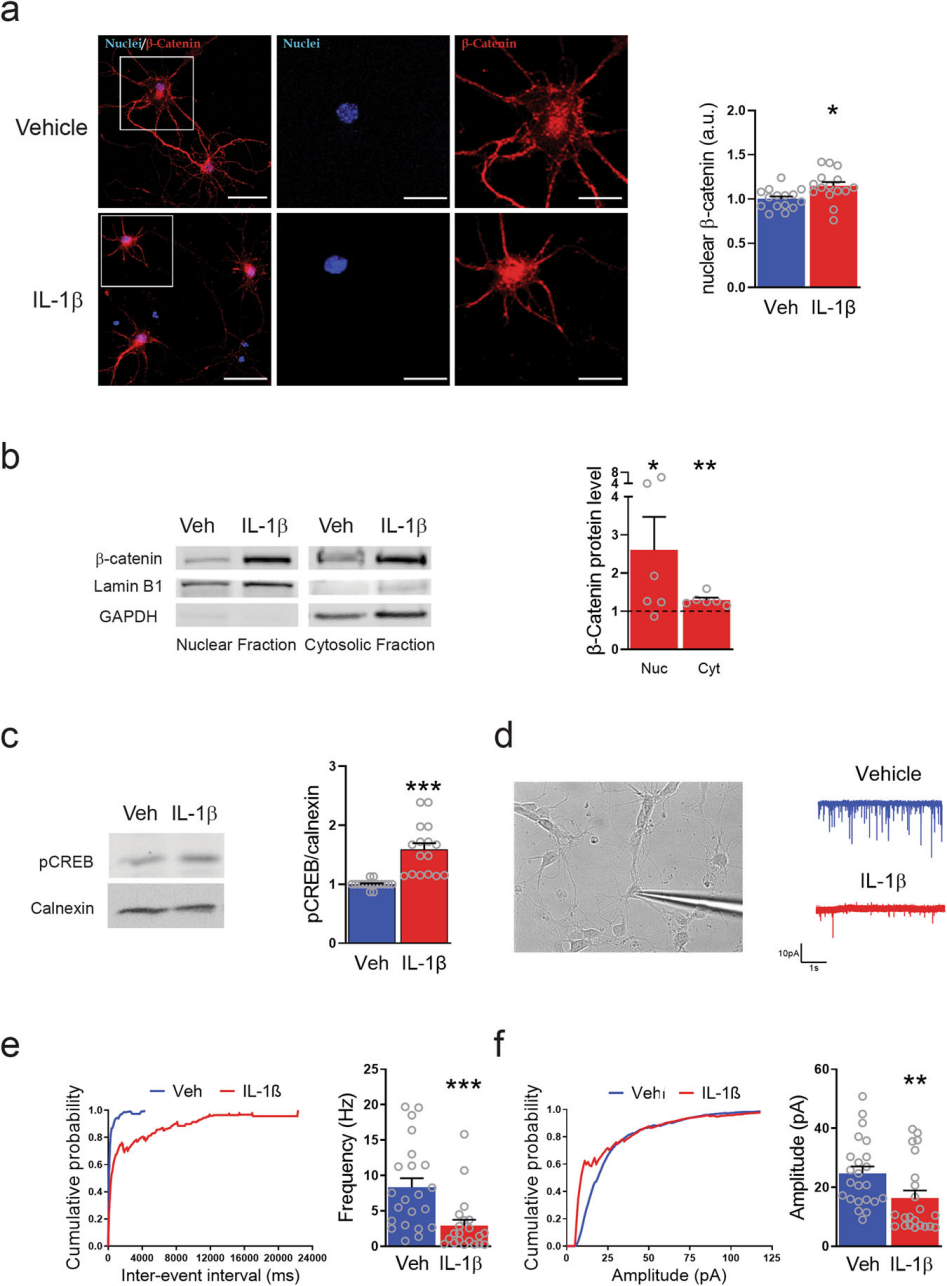
IL-1β treatment increases nuclear β-catenin and CREB activation, while it decreases excitatory synaptic strength in cortical neurons. **a** Representative images (left) and quantification (right) of nuclear β-catenin signal in primary neurons treated with either vehicle (Veh, blue bar) or IL-1β (red bar) for 24 h and stained for β-catenin (red). DAPI-stained nuclei are in blue. Separate channels are shown for the high-magnification images of the boxed regions. Scale bars, 50 and 10 μm for low and high magnification, respectively. **b** Representative immunoblots (left) and quantification (right) of β-catenin levels in nuclear and cytosolic fractions from primary neurons treated as in **a**. Immunoblotting for Lamin B1 and GAPDH as nuclear and cytosolic markers, respectively, was used to check the purity of the subcellular fractions. The changes in β-catenin levels in nuclear and cytosolic fractions upon treatment with IL-1β are expressed in percentage of the respective values in vehicle-treated samples. **c** Quantitative western blotting analysis of CREB phosphorylation in neurons treated with either IL-1β or vehicle (Veh) for 20 min. Calnexin was used as loading control. **d** Representative image of patch-clamped neurons (left) and representative traces of mEPSCs (right) recorded in cortical neurons treated with either vehicle (blue trace) or IL-1β (red trace) for 20 min at 7 DIV. Recordings were performed at 14 DIV. **e** Cumulative distribution of inter-event intervals (left) and mean (±sem) frequency (right) of mEPSCs. **f** Cumulative distribution (left) and mean (± sem) amplitude (right) of mEPSCs. Bar graphs show mean ± sem with superimposed individual points obtained from distinct culture dishes prepared from at least *n* = 3 independent preparations. Veh: *n* = 23 and IL-1β: *n* = 22. *p < 0.05, **p < 0.01, ***p < 0.001; unpaired two-tailed Student’s *t* test.

Given the involvement of Wnt/β-catenin signaling in neuroinflammation^57^ and the role of both Wnt/β-catenin and phospho-CREB in activating REST transcription^58,61^, we investigated their involvement in the signaling pathways triggered by IL-1β in neurons. When we analyzed β-catenin nuclear translocation by quantitative immunocytochemistry and western blotting of nuclear/cytoplasmic fractions, we found a significant increase in nuclear β-catenin levels in primary neurons upon IL-1β treatment, similar to the results obtained with the supernatant of activated T cells (Fig. 4a, b). We also evaluated the expression of the Ser-133-phosphorylated isoform of CREB (pCREB) in parallel with REST expression after 20-min treatment with IL-1β. Indeed, consistent with the notion that CREB signaling is activated by IL-1β, we found a significant increase in Ser-133-phosphorylated CREB, indicating that an activation of CREB signaling could be involved in the IL-1β-induced REST overexpression (Fig. 4c).

To gain some insights into the role of the IL-1β-induced REST response in synaptic strength and maintenance, the effects of the 20-min treatment with IL-1β applied at 7 DIV were evaluated by patch-clamp recordings in more mature neuronal networks in the presence of a developed synaptic connectivity at 14 DIV. We focused on miniature excitatory postsynaptic currents (mEPSCs), whose frequency and amplitude provide information on the density of synaptic connections and on the strength of individual synapses. Wild-type primary cortical neurons previously treated with IL-1β at 7 DIV displayed an impairment in the excitatory synaptic strength at 14 DIV, in the absence of any effect of IL-1β on neuronal viability (Supplementary Fig. 2). Indeed, we observed a significant decrease of both miniature excitatory postsynaptic potential (mEPSP) frequency (Fig. 4d, e) and amplitude (Fig. 4d, f). While mEPSC frequency reflects the density of synaptic inputs to the patched neuron and the probability of spontaneous release, mEPSC amplitude reflects the strength of synaptic transmission in response to the release of a single quantum of neurotransmitter. The results thus suggest a multifaceted impairment of synaptic transmission induced by IL-1β.

### The IL-1β-dependent downscaling of excitatory synaptic transmission is mediated by REST

To demonstrate that the downscaling of excitatory transmission induced by IL-1β was dependent on the transcriptional activation of REST, the same experiments were repeated in cortical neurons derived from REST^GTi/GTi^ mice, a model bearing a conditional gene trap (GTi) cassette in an intron of the endogenous REST gene^52^ (Supplementary Fig. 3a). In these neurons, REST depletion was induced by transduction with lentiviral particles encoding for Cre recombinase to terminate REST transcription (herein referred to as Cre-REST). Control neurons (herein referred to as ΔCre-REST) were transduced with an inactive form of Cre-recombinase (ΔCre-recombinase). Quantitative real-time PCR and western blotting analysis confirmed the effective downregulation of REST mRNA and protein, respectively (Supplementary Fig. 3b, c).

**Fig. 5:**
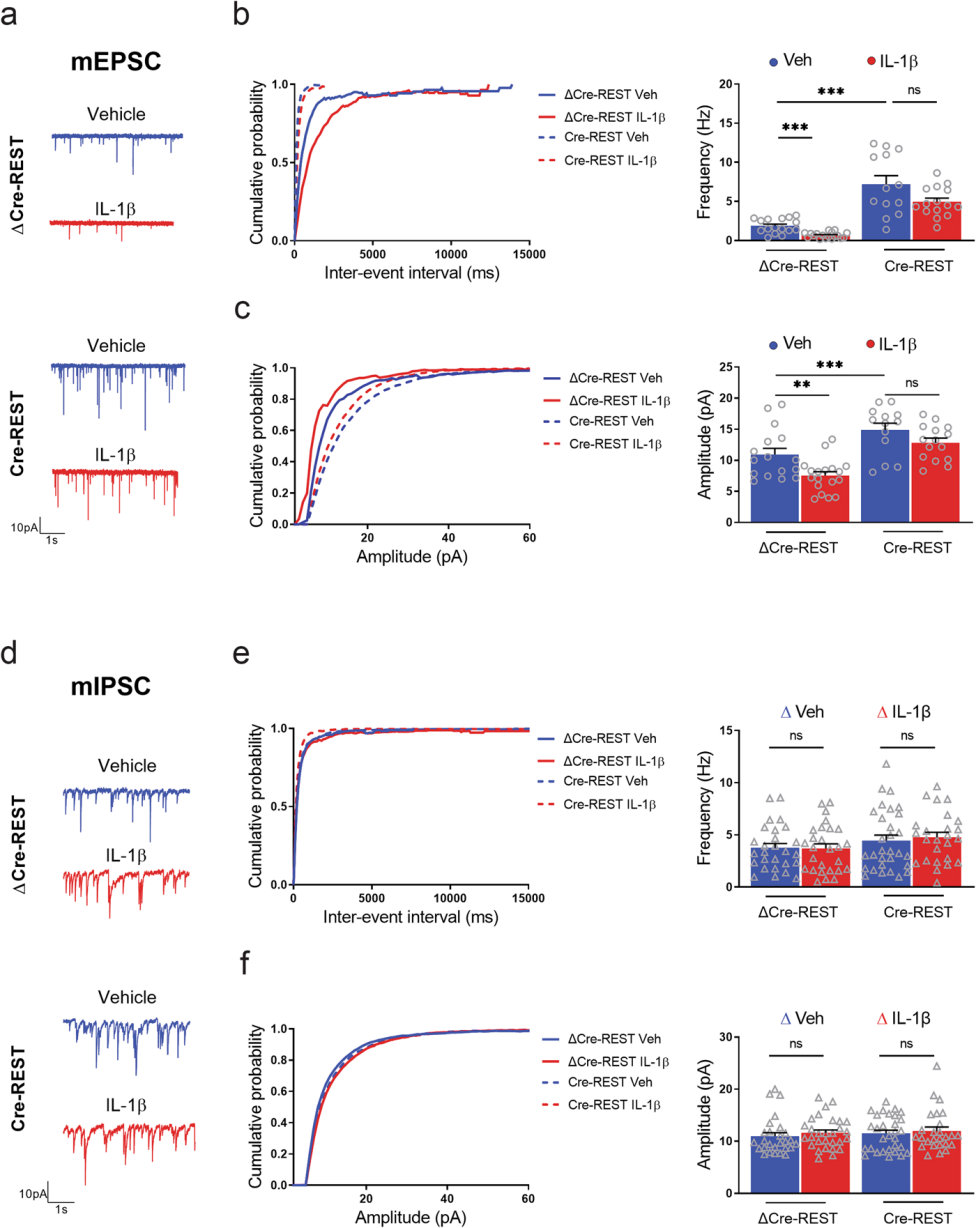
REST deletion occludes the effects of IL-1β treatment on excitatory transmission. **a**–**c** Representative traces (**a**), cumulative distribution of inter-event intervals (left) and mean frequency (right) (**b**), and cumulative distribution (left) and mean amplitude (right) (**c**) of mEPSCs in ΔCre-REST and Cre-REST transduced cortical neurons treated with either vehicle (blue traces) or IL-1β (red traces) for 20 min at 7 DIV. Recordings were performed at 14 DIV. **d**–**f** Representative traces (**d**), cumulative distribution of inter-event intervals and mean frequency (**e**), and cumulative distribution and mean amplitudes (**f**) of mIPSCs recorded under the same experimental conditions described above. Graphs show mean ± sem of at least three independent preparations with superimposed individual points. Excitatory synapses: *n* = 16, 18, 13, and 15; inhibitory synapses: *n* = 27, 29, 30, and 26 for ΔCRE-REST Veh, ΔCRE-REST IL-1β, CRE-REST Veh, and CRE-REST IL-1β, respectively. **p < 0.01, ***p < 0.001, ns: *p* > 0.05; two-way ANOVA/Bonferroni’s tests.

ΔCre-REST-transduced neurons treated with IL-1β showed a decrease in mEPSP frequency and amplitude similar to that observed in wild-type neurons (Fig. 5a–c) and were taken as controls for the effects of REST deletion. Untreated REST-knockout (REST-KO) neurons displayed an upscaling of excitatory synaptic transmission characterized by a significant increase in both frequency and amplitude of mEPSCs (Fig. 5a–c), consistent with previous reports^26^. However, treatment of these REST-KO neurons with IL-1β did not significantly alter the effects of REST deletion on mEPSC frequency (Fig. 5a, b) and amplitude (Fig. 5a, c), demonstrating that the REST deletion occluded the effects of IL-1β on synaptic transmission.

We performed the same type of analysis on inhibitory synapses by studying frequency and amplitude of miniature inhibitory postsynaptic currents (mIPSCs). Interestingly, the results showed that IL-1β did not affect either frequency or amplitude of mIPSCs in control neurons transduced with ΔCre (Fig. 5d–f), indicating that the cytokine-induced synaptic scaling was specific for excitatory synapses. The same phenomenon was observed when REST was knocked out in neurons transduced with active Cre-recombinase, leaving mIPSC frequency and amplitude unchanged with respect to untreated REST-expressing neurons (Fig. 5d–f). Again, when the IL-1β treatment was applied in neurons knocked out for REST, no further change in mIPSC frequency or amplitude was observed with respect to untreated REST-KO neurons (Fig. 5d–f).

### IL-1β decreases the density of excitatory synapses and the frequency of calcium oscillations through REST upregulation

**Fig. 6:**
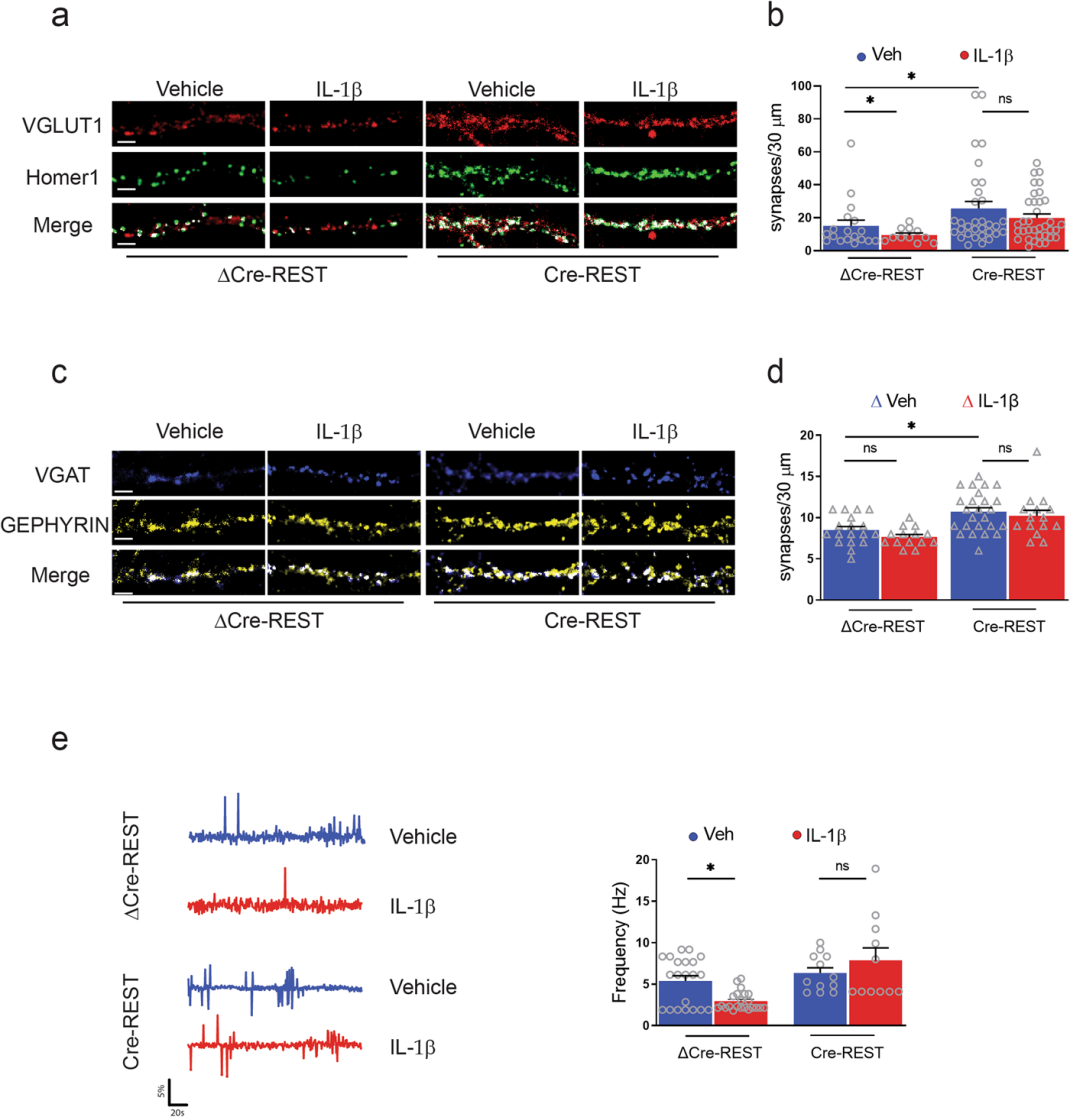
REST deletion occludes the IL-1β-mediated decrease in the density of excitatory synapses. **a**, **b** Excitatory synapses. **a** Representative images of excitatory synaptic boutons in proximal dendrites from ΔCre- and Cre-infected neurons treated with either vehicle or IL-1β for 20 min at 7 DIV and analyzed at 14 DIV. Excitatory synapses were identified by double immunostaining with VGLUT1/Homer1. The merged panels highlight the double-positive puncta (white) corresponding to bona fide synapses. **b** Quantification of the linear density of inhibitory synapses expressed as the mean number of excitatory boutons counted on 30-µm dendritic branches starting from the cell body in ΔCre- and Cre-infected neurons treated with vehicle (veh; blue bars) or IL-1β (red bars). **c**, **d** Inhibitory synapses. **c** Representative images of inhibitory synaptic boutons identified by double immunostaining with VGAT/Gephyrin under the same experimental conditions shown in **a**. **d** Quantification of the linear density of inhibitory synapses expressed as the number of inhibitory boutons counted on 30-µm dendritic branches starting from the cell body in ΔCre- and Cre-infected neurons treated with vehicle (veh; blue bars) or IL-1β (red bars). Scale bars, 6 µm. **e** Representative traces (left) and frequency of calcium oscillations (right) in ΔCre-REST and Cre-REST transduced cortical neurons treated with either vehicle (blue traces) or IL-1β (red traces) for 20 min at 7 DIV. Recordings were performed at 14 DIV. Peaks with at least 2% of difference with respect to the baseline were considered and their frequency was calculated as total number of significant peaks over the recording time. Bar graphs in **b**, **d**, **e** show mean ± sem with superimposed individual points from independent coverslips prepared from at least *n* = 3 distinct preparations. *p < 0.05; two-way ANOVA/Bonferroni’s tests.

The specific decrease induced by IL-1β in the frequency of mEPSCs could in principle be attributed to a decreased probability of spontaneous release or to a change in the density of synaptic connection impinging on the recorded neuron. To test these possibilities, we performed double immunofluorescence staining of presynaptic and postsynaptic markers to unambiguously identify excitatory and inhibitory synaptic boutons. Excitatory synapses were stained for the presynaptic VGLUT1 and the postsynaptic scaffold protein Homer (Fig. 6a), while inhibitory synapses were labeled for the presynaptic VGAT and the postsynaptic scaffold protein Gephyrin (Fig. 6c). Consistent with the electrophysiological data, the treatment with IL-1β significantly and specifically decreased the density of excitatory synapses in control neurons transduced with ΔCre (Fig. 6b), confirming the basis for the decrease in mEPSC frequency and underlining the specificity of the IL-1β effects for excitatory synapses. At the same time, no effects of IL-1β on the density of inhibitory synapses were observed, as could be predicted by the unaffected inhibitory synaptic currents (Fig. 6d). Cre-mediated REST deletion was followed by an increase in excitatory (Fig. 6a, b) and, to a lesser extent, inhibitory (Fig. 6c, d) synaptic puncta. However, when REST-KO neurons were subjected to IL-1β treatment, the decrease in the number of excitatory synapses observed in control neurons was virtually abolished (Fig. 6a, b), indicating that the cytokine effect was fully occluded by REST deletion. As observed in control neurons, IL-1β treatment did not modify the density of inhibitory synaptic puncta in neurons in which REST was knocked out (Fig. 6c, d). We also analyzed the REST dependence of the IL-1β effects on the excitability of neuronal networks by Fura-2 AM live calcium imaging. Spontaneous calcium oscillations reflect spontaneous neuronal firing, which in turn depends on both intrinsic excitability and strength of excitatory transmission. Coherently with what observed in excitatory synaptic transmission, IL-1β decreased the frequency of spontaneous calcium transients in normal neurons, while it failed to affect calcium oscillations in neurons that had been knocked out for REST (Fig. 6e). Taken together, the results demonstrate that the IL-1β-induced reduction of excitatory synaptic contacts and excitatory strength is entirely dependent on REST-mediated transcriptional regulation.

## Discussion

The process of neuroinflammation is a shared feature of several pathologies and is associated with synapse dysfunction and cognitive decline in human patients and animal models. Among the numerous diseases associated with inflammation, MS presents a severe and progressive inflammatory reaction in the central nervous system. Genetic, histochemical, and biochemical studies of postmortem brains have demonstrated synaptic loss in patients with MS, as well as in rodents with EAE^62^. In this respect, a role for pro-inflammatory cytokines released by activated microglia/macrophages and infiltrating T lymphocytes has been proposed based on in vivo and in vitro studies. Although it is long known that neuroinflammation impacts on synaptic function and that MS implies a chronic phase of synaptic deficits, the molecular mechanisms and signaling maps implicated in immune-synaptopathies are only partly uncovered. Cytokines activate intracellular signaling pathways targeting the activity of multiple transcription factors, thus modifying the cellular gene transcription profile. One of the most important transcription factors modulating neural function is REST/NRSF that, by binding to RE-1 elements present in the promoter region of a huge cluster of neural genes, markedly modifies the neuronal phenotype, neuronal excitability, and synaptic transmission.

In this paper, we investigated whether REST is among the transcription factors activated by neuroinflammation. Given that REST acts as a transcriptional repressor of neural genes, including the vast majority of synaptic proteins, its activation could easily explain the synaptic impairment observed during neuroinflammation. We found that REST is activated by inflammation in both neuroblastoma cells and primary neurons, indicating that a vast transcriptional change—common to different neural cell types—is triggered by inflammatory stimuli. Our data show that REST transcriptional activation occurs upon a variety of neuroinflammatory stimuli. Indeed, such transcriptional activation of REST was observed upon treatment with activated T cell supernatant, known to include a variety of pro-inflammatory cytokines among which IL-2, TNF-α, and IFN-γ, after exposure to LPS-activated microglia, and upon exposure to purified IL-1β, one of the major inflammatory cytokines produced by activated microglia as a result of neuroinflammation^63–65^.

A second relevant point is that the expression of the splicing isoform REST4 was found to depend on the type of target cells. Indeed, in differentiated N2a cells a dual activation of REST and REST4 was observed, consistent with the increased expression of nSR100, one of the factors involved in regulating REST/REST4 splicing. On the other hand, primary neurons responded with a selective full-length REST upregulation in the absence of changes in REST4/nSR100 expression. In both cases, however, the upregulation of REST/REST4 was associated with activation of Wnt signaling and increased nuclear translocation of β-catenin, a well-known intracellular transduction pathway in neuroinflammation.

Recent data^66^ have shown that inflammation, and in particular the pro-inflammatory cytokine IL-1β, negatively impacts on synapse structure and function by specifically affecting synaptic genes^59^. Evidence from several studies suggests that IL-1β positively and/or negatively regulates voltage- and ligand-gated neuronal ion channel activity^67,68^. IL-1β is a potent inflammatory cytokine that acts as an important mediator of neuronal injury^69^. Indeed, it is involved in the pathogenesis and outcome of several brain diseases, including MS^70^, Alzheimer’s disease^71^, epilepsy^72^, stroke^73^, and neurodevelopmental disorders^74,75^. In line with this experimental evidence, we found that, among several tested cytokines, IL-1β selectively caused a potent and prompt increase in REST transcription and translation in neurons, associated with increased CREB phosphorylation that is known to play a role in activating REST transcription. Remarkably, IL-1β activation in neurons brought about a delayed and strong synaptic downscaling that was specific for excitatory synapses and that impaired both the frequency and the amplitude of spontaneous synaptic currents, as well as the frequency of spontaneous calcium oscillations that are a proxy of action potential firing. These observations suggested a presynaptic/postsynaptic level of action, which was in fact supported by the decreased density of synaptic connections. Interestingly, IL-1β activation left inhibitory synapses unaffected. Importantly, the IL-1β effects on excitatory transmission and calcium oscillations were strictly REST dependent, as the Cre-mediated deletion of REST completely occluded the synaptic effects of IL-1β activation, indicating that REST upregulation is necessary and sufficient for the downscaling of neuronal activity induced by IL-1β.

## Conclusions

In conclusion, the increased transcriptional repression induced by pro-inflammatory cytokines involves the activation of REST repressor response. Whether this response represents a compensatory homeostatic mechanism in response to immunological stress or whether the same is pathogenic and responsible for the synapse dysfunctions and cognitive impairments associated with neuroinflammation is a very interesting topic for further investigations. Future studies will be necessary to determine whether sustained IL-1β activation could also recapitulate the acute effects described in this study. The possibility of selectively inhibiting IL-1β signaling may allow to dissect the physio-pathological role of the synaptic scaling that characterize immuno-synaptopathies, with the aim of devising therapeutic strategies to ameliorate the clinical outcome.

## Supplementary information

Supplemental figures and legends

## Data Availability

The datasets used and/or analyzed during the current study are available from the corresponding author on reasonable request.
