# Correction: Neuroinflammation induces synaptic scaling through IL-1β-mediated activation of the transcriptional repressor REST/NRSF

**DOI:** 10.1038/s41419-023-05805-0

**Published:** 2023-05-08

**Authors:** Federica Buffolo, Valentina Petrosino, Martina Albini, Matteo Moschetta, Federico Carlini, Thomas Floss, Nicole Kerlero de Rosbo, Fabrizia Cesca, Anna Rocchi, Antonio Uccelli, Fabio Benfenati

**Affiliations:** 1 Center for Synaptic Neuroscience and Technology, Istituto Italiano di Tecnologia, Largo Rosanna Benzi 10, 16132 Genova, Italy; 2 Department of Experimental Medicine, University of Genova, Viale Benedetto XV, 3, 16132 Genova, Italy; 3 Department of Neurosciences, Rehabilitation, Ophthalmology, Genetics, Maternal and Child Health, University of Genova, Largo P. Daneo, 3, 16132 Genova, Italy; 4 IRCCS, Ospedale Policlinico San Martino, Largo Rosanna Benzi 10, 16132 Genova, Italy; 5 Helmholtz Zentrum München, Deutsches Forschungszentrum für Gesundheit und Umwelt (GmbH), Ingolstädter Landstr. 1, 85764 Neuherberg, Germany; 6 Department of Life Sciences, University of Trieste, Trieste, 34127 Italy

**Keywords:** Cytokines, Cellular neuroscience

Correction to: *Cell Death and Disease* 10.1038/s41419-021-03465-6, published online 15 February 2021

The original version of this article contained an error in the funding section. The correct funding should read: “This work was supported by Fondazione Italiana Sclerosi Multipla (FISM project # 1946 to AU and F. Benfenati), Compagnia di San Paolo Torino (no. 34760 to F. Benfenati), Ministero Istruzione, Università e Ricerca (PRIN-2017A9MK4R to F. Benfenati), Ministero della Salute (Ricerca Corrente to FB; Ricerca Finalizzata Giovani Ricercatori GR-2019-12370176 to AR)”. The authors apologize for the error. The original article has been corrected.

