# Supplementary material for: Neuroinflammation induces synaptic scaling through IL-1β-mediated activation of the transcriptional repressor REST/NRSF
Source: Cell Death Dis. 2021 Feb 15;12(2):180. doi: 10.1038/s41419-021-03465-6 (PMC7884694; doi:10.1038/s41419-021-03465-6)
Supplement: Supplementary file 1 — Supplemental figures and legends [file 41419_2021_3465_MOESM1_ESM.docx]

*Cell Death and Disease*

Manuscript CDDIS-20-3567RR

Supplementary Materials

**Neuroinflammation induces synaptic scaling through IL-1β-mediated activation of the transcriptional repressor REST/NRSF**

F. Buffolo^1,2^*, V. Petrosino^3,4^*, M. Albini^1,2^*, M. Moschetta^1,4^, F. Carlini^3,4^, T. Floss^5^, N. Kerlero de Rosbo^3^, F. Cesca^1,6^, A. Rocchi^1,4§^, A. Uccelli^3,4^*^§^, F. Benfenati^1,4^*^§^

^1^ Center for Synaptic Neuroscience and Technology, Istituto Italiano di Tecnologia, Largo Rosanna Benzi 10, 16132 Genova, Italy; ^2^ Department of Experimental Medicine, University of Genova, Viale Benedetto XV, 3, 16132 Genova, Italy; ^3^ Department of Neurosciences, Rehabilitation, Ophthalmology, Genetics, Maternal and Child Health, University of Genova, Largo P. Daneo, 3, 16132 Genova, Italy; ^4^ IRCCS, Ospedale Policlinico San Martino, Largo Rosanna Benzi 10, 16132 Genova, Italy; ^5^ Helmholtz Zentrum München, Deutsches Forschungszentrum für Gesundheit und Umwelt (GmbH), Ingolstädter Landstr. 1, 85764 Neuherberg, Germany; ^6^ Department of Life Sciences, University of Trieste, Trieste, Italy.

* These authors contributed equally to this work

^§^ Corresponding authors

**SUPPLEMENTARY MATERIALS**

**
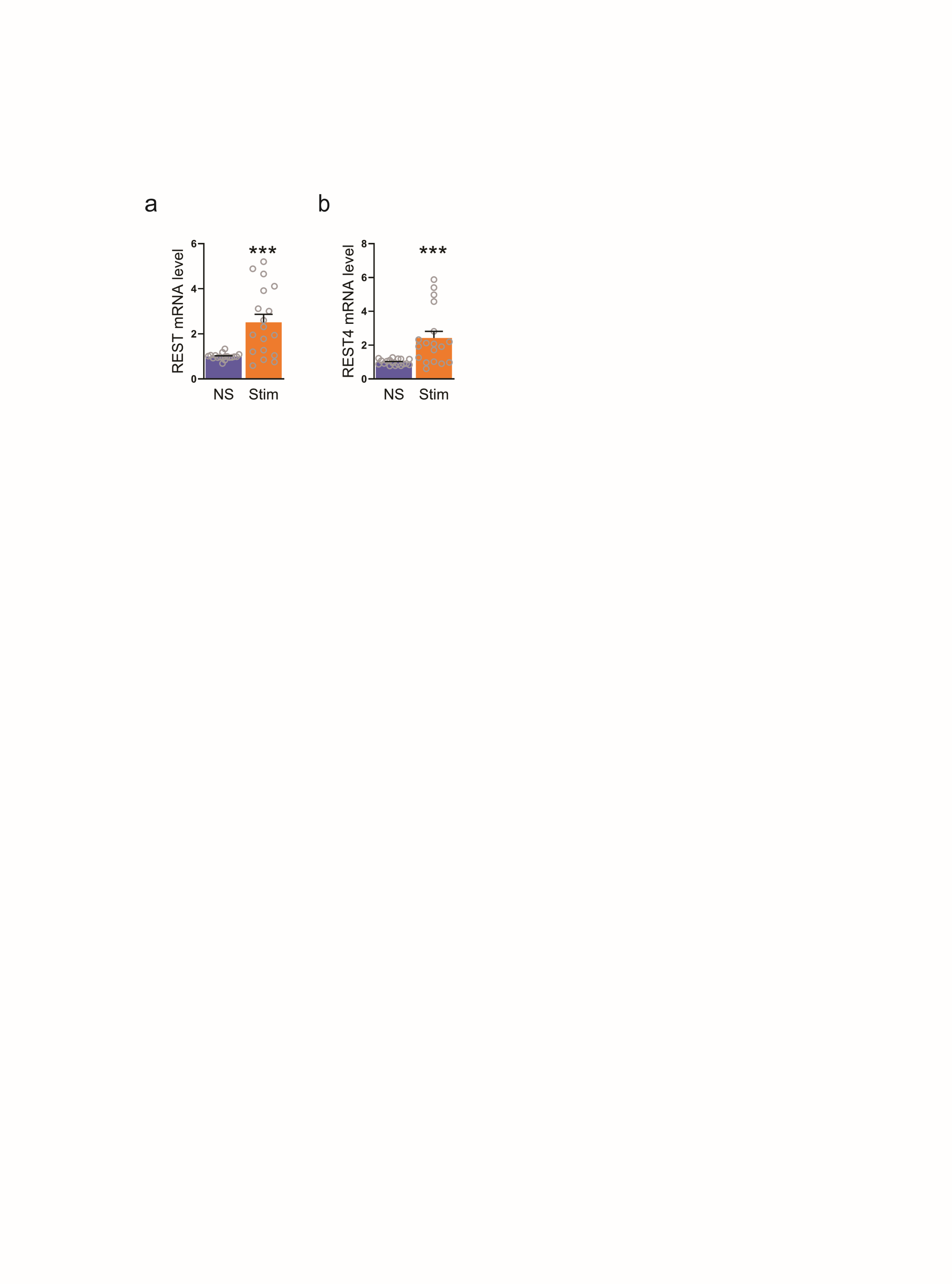
**

**Supplementary Figure 1. IL-1β treatment increases REST and REST 4 mRNAs in N2a cells.** The mRNA levels of REST **(a)** and REST4 **(b)** were quantified by qRT-PCR in IL-1β- treated-cells as compared to control cells (NS). Gapdh, Actin and Hprt1 were used as housekeeping genes in qRT-PCR analyses Graphs show means ± sem with superimposed individual points from distinct culture dishes prepared from at least n=3 independent preparations. ***p<0.001; Mann-Whitney *U*-test.

**
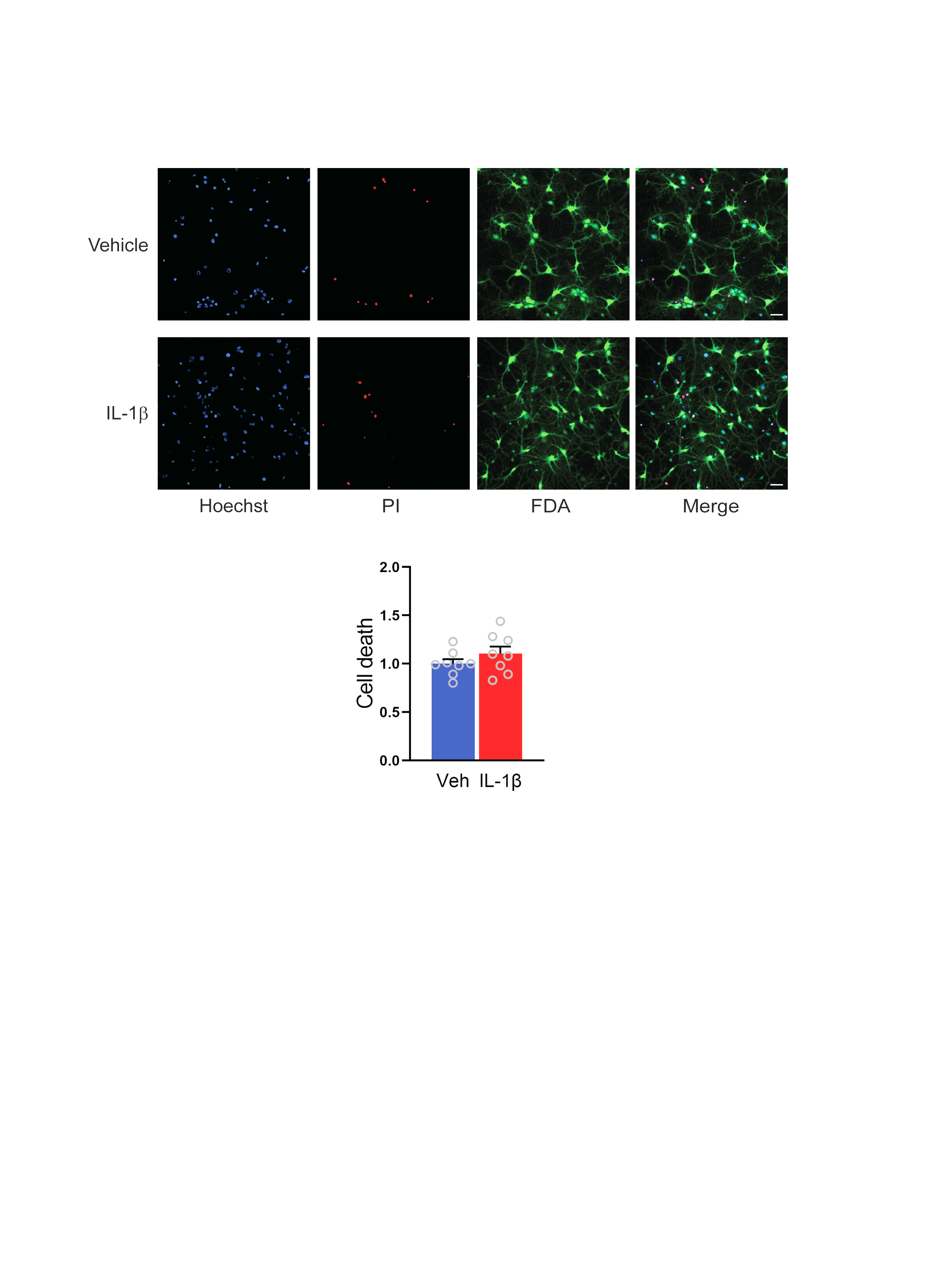
**

**Supplementary Figure 2. Neuronal cell viability after 7 days upon exposure to IL-1β.** Primary mouse cortical neurons were exposed to IL-1β (20 ng/ml) or vehicle (veh; 0.1% BSA) for 20 min at 7 DIV and analyzed at 14 DIV. Cell viability was evaluated by fluorescence microscopy. Representative images of neuronal cultures stained with Hoechst 33342 for nuclear visualization (blue), propidium iodide (PI; red) for cell death quantification and fluorescein diacetate (FDA; green) for cell viability detection. Scale bars, 100 μm. The percentages of PI-positive cells with respect to the total number of Hoechst-positive cells, were calculated for each experimental group and normalized to the values of vehicle-treated samples. The bar plot shows the means ± sem with superimposed individual points from distinct culture dishes prepared from at least n=3 independent preparations. p>0.05; unpaired two-tailed Student’s *t*-test.

**
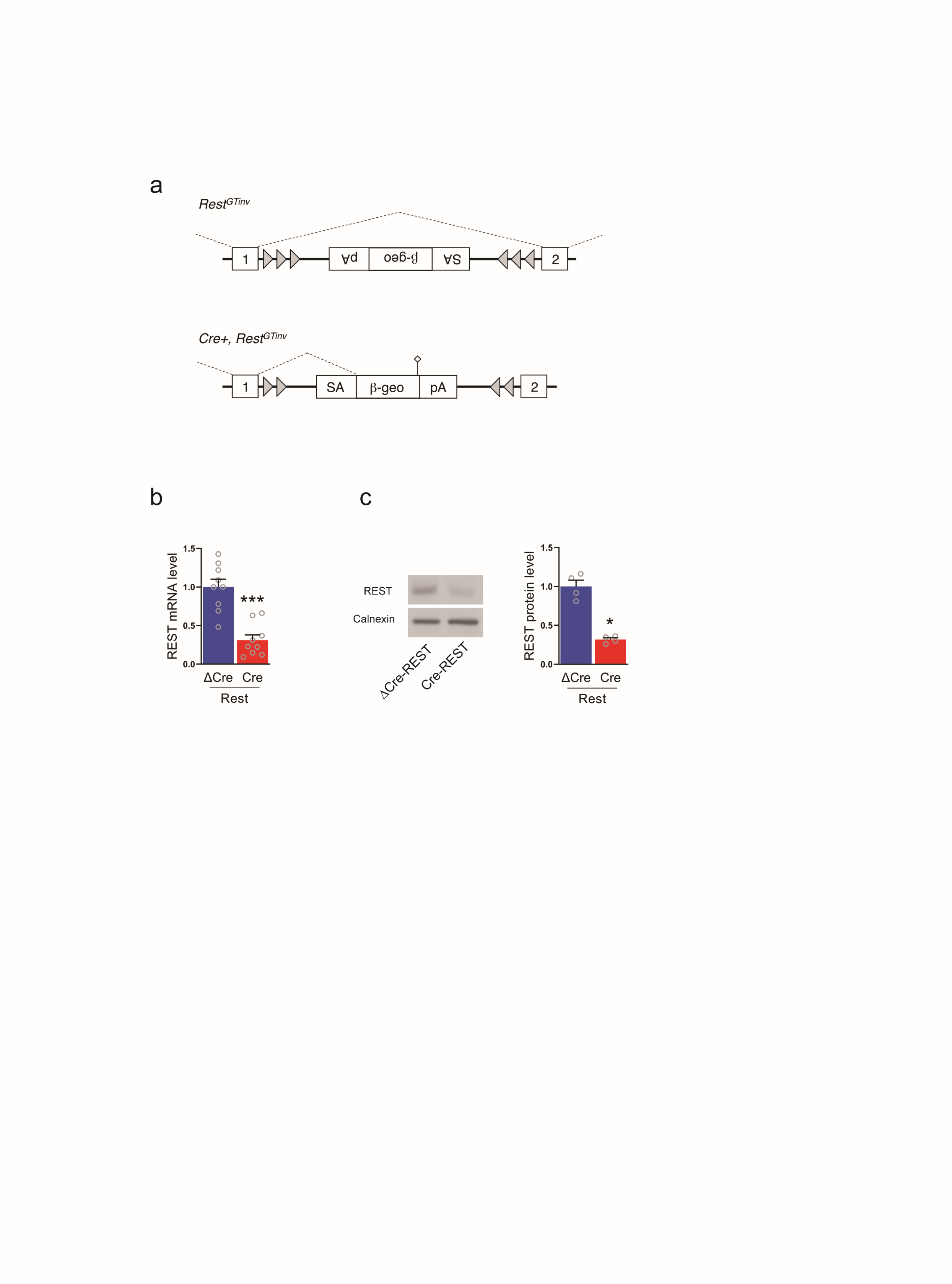
Supplementary Figure 3. Characterization of REST silencing in REST^GTi^ cortical neurons. (a)** Schematic representation of REST GTinv cassette, modified from (52). **(b,c)** Primary cortical neurons from REST^GTi^ mice were transduced with lentiviral vectors encoding defective (ΔCre) and active (Cre) Cre-recombinase and the expression of full-length REST was assessed by qRT-PCR (b) and western blotting (c) analysis. In (c), a representative experiment and the respective quantification are shown. The residual levels of REST mRNA/protein reflect the near complete transduction of primary neurons. In qRT-PCR analysis Actin, Gusb and Gapdh were used as reference genes; in Western blotting analysis, Calnexin was used as a loading control. Graphs show means ± sem with superimposed experimental points (n=5-9). *p<0.05, ***p<0.001; unpaired two-tailed Student’s *t*-test.
